# Dual targeting of PI3Kδ and PPARα enhances antitumor activity via FoxO1 activation in follicular lymphoma

**DOI:** 10.1038/s41419-026-08593-5

**Published:** 2026-03-23

**Authors:** Wenqin Wang, Hui Zhou, Shuangxiong Tan, Dongmei Qin, Shuxuan Wang, Chunlan Xu, Xiangru Lei, Wenjuan Li, Liangjie Wang, Shuhui Fu, Shuman Jia, Bing Xu, Jie Zha

**Affiliations:** 1 Department of Hematology, The First Affiliated Hospital of Xiamen University and Institute of Hematology, School of Medicine, Xiamen University, Xiamen, China; 2 Key Laboratory of Xiamen for Diagnosis and Treatment of Hematological Malignancy, Xiamen, 361003 China; 3 State Key Laboratory of Cellular Stress Biology, Innovation Center for Cell Biology, School of Life Sciences, Xiamen University, Xiamen, China

**Keywords:** Lymphoma, Cell death

## Abstract

Although phosphoinositide 3-kinase-δ (PI3Kδ) inhibition demonstrates efficacy in relapsed/refractory follicular lymphoma (FL), its clinical benefit is often limited by adaptive resistance, underscoring the need for rational combination strategies. Here, we show that combining the PI3Kδ inhibitor linperlisib with the pan-peroxisome proliferator-activated receptor (PPAR) agonist chiglitazar, an agent that reprograms tumor metabolism, delivers robust antitumor activity across FL models, including cell-derived and patient-derived xenografts, with a favorable tolerability profile. The combined regimen promotes G1/S arrest and apoptosis, exerting complementary metabolic and signaling effects through glycolysis suppression, activation of PPARα-driven programs, and consequent reactivation of the transcription factor forkhead box protein O1 (FoxO1), which is repressed by PI3K/AKT signaling. Genetic depletion of FoxO1 attenuates treatment responses, identifying FoxO1 activity as both a pharmacodynamic biomarker and a potential predictor of therapeutic benefit. Compared with monotherapy, the combination consistently achieves superior tumor control in vivo without overt toxicity, supporting its clinical translation potential. Collectively, these data provide a mechanistic rationale for dual targeting of PI3Kδ and PPARα in FL and advocate for clinical evaluation of this combination with FoxO1 as a pharmacodynamic biomarker.

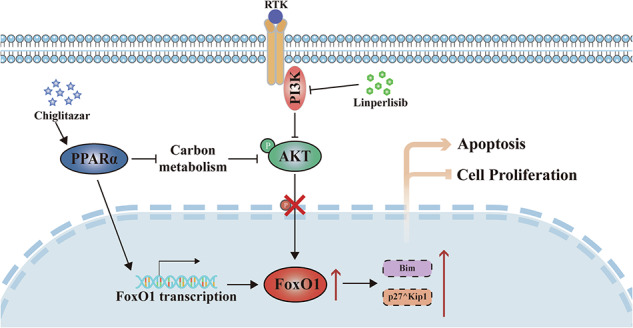

## Introduction

Follicular lymphoma (FL) is the second most common non-Hodgkin lymphoma subtype, usually following an indolent clinical course. Although frontline chemoimmunotherapy frequently achieves high initial response rates, the disease course is dominated by inevitable relapse occurring in over 50% of patients within 10 years, and carries an annual 2–3% risk of transformation to aggressive diffuse large B-cell lymphoma (DLBCL) [1–3]. These ongoing clinical challenges highlight the urgent need to develop new, mechanism-based therapeutic approaches [4].

Targeting key transcriptional nodes that govern lymphoma cell survival and metabolism presents a promising approach. The forkhead box O1 (FoxO1) transcription factor, a downstream effector of the phosphoinositide 3-kinase/Akt (PI3K/AKT) signaling pathway, critically regulates apoptosis, cell cycle progression, and cellular metabolism [5]. In FL and other B-cell lymphomas, FoxO1 is often inactivated through AKT-mediated phosphorylation, leading to its cytoplasmic sequestration and functional impairment [6–8]. The loss of FoxO1 activity correlated with diminished pro-apoptotic gene expression, enhanced tumor cell survival, and poorer patient outcomes [9, 10]. establishing it as a compelling therapeutic target.

The PI3Kδ inhibitor linperlisib has emerged as a promising therapeutic agent for relapsed or refractory FL by disrupting the PI3K/AKT signaling, which is essential for B-cell receptor signaling and lymphomagenesis [11–15]. However, the therapeutic potential of linperlisib monotherapy is frequently constrained by transient response durations and the development of acquired resistance. These limitations primarily stem from compensatory feedback mechanisms and cellular adaptation processes that enable tumor survival despite PI3Kδ inhibition [16]. Chiglitazar, a pan-peroxisome proliferator-activated receptor (PPAR) agonist with potent PPARα activity, can reprogram tumor cell metabolism by promoting oxidative phosphorylation and suppressing glycolysis [17–19]. Given that metabolic plasticity is a recognized hallmark of FL, exploiting this vulnerability may provide an effective complement to PI3K inhibition [20–23]. Furthermore, emerging preclinical evidence suggests PPAR activation can modulate upstream signaling pathways, including PI3K/AKT, thereby potentially influencing FoxO1 activity [24].

Based on this rationale, we hypothesized that dual targeting of oncogenic signaling and metabolic dependencies through the combination of linperlisib and chiglitazar would yield synergistic antitumor activity in FL models. We further postulated that FoxO1 reactivation would serve as a critical integrative mechanism linking PI3Kδ inhibition to metabolic reprogramming. This study was designed to evaluate the therapeutic efficacy and mechanistic basis of this combination in FL models in vitro and in vivo. Our findings demonstrate that co-targeting PI3Kδ and PPARα results in robust inhibition of FL cell proliferation, induction of apoptosis, and cell cycle arrest. Mechanistically, these effects converge on the functional restoration of FoxO1. These results provide a strong mechanistic foundation for the clinical translation of this combination strategy in FL.

## Methods

### Reagents and cell culture

Linperlisib was commercially sourced from YL-PHARMA Ltd. (Shanghai, China), whereas chiglitazar was procured from Chipscreen Bioscience Ltd. (Shenzhen, China). The RL, Karpas-422, and Sc-1 cell lines were maintained in our laboratory and cultured in Roswell Park Memorial Institute (RPMI)-1640 medium (HyClone, Thermo Scientific, MA, USA) supplemented with 10% fetal bovine serum (FBS), 100 μg/mL streptomycin, and 100 U/mL penicillin. Cells were cultured at 37 °C in a humidified atmosphere with 5% CO_2_. HEK293T cells were obtained from ATCC (Teddington, UK) and cultured in Dulbecco’s Modified Eagle Medium under identical conditions.

### Cell proliferation and viability assays

RL, Karpas-422, and Sc-1 cells were seeded at a density of 2 × 10^5^ cells/mL, treated with dimethyl sulfoxide (DMSO; control) or linperlisib and chiglitazar (alone or in combination) at specified concentrations for 24 h, and then counted at various time points via trypan blue exclusion (MCE, Shanghai, China). For viability assays, 1 × 10^4^ cells/well were plated in 96-well plates with 100 µL medium, exposed to the designated drug treatments for 24 h, and assessed using a Cell Counting Kit-8 (CCK-8) kit (MCE).

The fraction affected (Fa) and combination index (CI) were calculated using CompuSyn software according to the Chou–Talalay median effect method [25]. and Fa–CI curves were generated to evaluate synergism or antagonism.

### Animal models

Cell-derived xenograft (CDX) models were established using RL cells in 6–7-week-old CB17 severe combined immunodeficiency (SCID) mice (*n* = 5, 6 per group). Before tumor cell inoculation, the mice received 1.5 Gy total body irradiation. Subsequently, 1 × 10^7^ RL cells were subcutaneously injected into the right flank. Three days after implantation (designated as day 1), the mice were treated by daily oral gavage for 14 consecutive days with linperlisib (60 mg/kg) and chiglitazar (10 mg/kg). Tumor growth was monitored every other day using caliper measurements, and tumor volume was calculated using the following formula: volume = (length × width^2^)/2. Therapeutic efficacy was assessed via tumor growth inhibition rate and survival analyses.

Patient-derived xenograft (PDX) models were generated using primary tumor specimens obtained from patients with FL, following the same treatment regimen as described for the CDX models.

All animal studies were approved by the Animal Care and Use Committee of Xiamen University (Approval No. XMULAC20220099) and performed in accordance with institutional guidelines and the ARRIVE reporting standards.

### Flow cytometric analysis of apoptosis

To evaluate drug-induced apoptosis, RL, Karpas-422, and Sc-1 cells (5 × 10⁵ cells/well) were seeded in 24-well plates with 1 mL complete medium and treated with the indicated compounds for 24 h, followed by staining with Annexin V/PI (Biolegend) in dark for 15 min. Then, flow cytometry was used to analyze the percentage of apoptotic cells (Annexin V^+^).

### 5-ethynyl-2′-deoxyuridine (EdU) assay

Following 24 h of treatment (as described above), the cells were washed with PBS and incubated with 10 µM EdU in fresh RPMI-1640 medium for 2 h. EdU incorporation was assessed using the BeyoClick™ EdU Cell Proliferation Kit with Alexa Fluor 594 (C0078S, Beyotime Biotechnology), as per the manufacturer’s protocol.

### Cell cycle analysis by flow cytometry

Cells were pretreated with 500 ng/mL nocodazole for 16 h to achieve cell cycle synchronization. After 48 h of drug treatment (as described above), the cells were harvested, washed with PBS, and fixed in 70% ethanol at −20 °C overnight. Fixed cells were then incubated with 1.0 mg/mL RNase A in PBS at 37 °C for 30 min, followed by staining at 4 °C for 20 min with 50 µg/mL propidium iodide (PI; BD Biosciences, San Jose, CA, USA) in PBS containing 0.1% Triton X-100. Cell cycle distribution was analyzed via flow cytometry.

### Glucose uptake

Following 24 h of drug treatment (as described above), the cells were incubated with 200 µM 2-NBDG (2-[N-(7-nitrobenz-2-oxa-1,3-diazol-4-yl) amino]-2-deoxy-D-glucose; HY-116215, MedChemExpress) in PBS at 37 °C for 40 min to assess glucose uptake. After washing with ice-cold PBS, the cells were stained with surface marker antibodies for 30 min at 4 °C, followed by another PBS wash. 2-NBDG fluorescence intensity was immediately analyzed by flow cytometry.

### RNA sequencing

For transcriptome profiling, 1 × 10^6^ RL cells were treated with linperlisib (32 μM) and chiglitazar (16 μM) for 24 h. Total RNA was isolated using TRIzol reagent (Invitrogen). RNA sequencing libraries were prepared and subjected to high-throughput sequencing (150 bp paired-end) by HaploX Biotechnology (Shenzhen, China). Raw data were processed using the standard bioinformatics pipeline for quality control, alignment, and differential gene expression analysis.

### RNA isolation and qPCR

RL, Karpas-422, and Sc-1 cells were seeded in 6-cm plates (1 × 10^6^ cells/plate) containing 4 mL complete medium and treated for 24 h with DMSO (control), 32 μM linperlisib, 16 μM chiglitazar, or both drugs in combination. Total RNA was isolated using TRIzol reagent (Invitrogen), and 1 μg RNA was reverse-transcribed into cDNA using the Evo M-MLV RT Kit (Accurate Biotechnology, Hunan, China). Quantitative PCR was performed using ChamQ Universal SYBR qPCR Master Mix (Vazyme Biotech) on a Bio-Rad CFX96 real-time PCR system, with relative gene expression quantified by the 2^−ΔΔCT^ method using β-actin as the endogenous control. The primer sequences are listed in Table S1.

### Metabolomic profiling

For metabolomic analysis, 1 × 10^7^ RL cells were treated with linperlisib (32 μM) and chiglitazar (16 μM) for 24 h. Cells were then harvested, and metabolites were extracted using a methanol-based protocol. Metabolomic profiling was conducted by BIOTREE (Shanghai, China) using liquid chromatography-mass spectrometry (LC–MS). Raw data were processed through a standard metabolomics data analysis pipeline, including peak detection, alignment, normalization, and identification of differentially abundant metabolites.

### FoxO1 knockdown cell line establishment

Two RL cell lines with stable FoxO1 knockdown (sh*FoxO1*#1 and sh*FoxO1*#2) were established via lentiviral transduction. Short hairpin RNA (shRNA) sequences targeting human *FoxO1* (based on NM_002015.4) were cloned into the pLVX-shRNA2-Puro vector (purchased from TsingKe Biological Technology). Lentiviral particles were produced in HEK293T cells using standard packaging systems and subsequently transduced into RL cells. After puromycin selection, stably transduced cells were maintained and validated by qPCR and western blotting to confirm *FoxO1*-knockdown efficiency. The shRNA sequences used are listed in Table S1.

### Western blot analysis

Western blotting was performed as per standard protocols, as previously described [26]. Cytoplasmic and nuclear proteins were extracted using the NE-PER® Extraction Kit (Thermo Scientific). The primary antibodies used were as follows: PI3K p110δ (A19742; ABclonal), pan-Akt (#4691; CST), phospho-Akt (Ser473, #4060; CST), PPARα (ab227074; Abcam), FoxO1 (#2880; CST), phospho-FoxO1 (Thr24, #9464; CST) and (Ser256, #9461; CST), FoxO3a (#12829; CST), FoxO4 (#9472; CST), Mcl-1 (#16225-1-AP; Proteintech), Bcl-2 (#12789-1-AP; Proteintech), Bim (A19702; ABclonal), Bax (#50599-2-Ig; Proteintech), Cleaved PARP(#5625; CST), P21(#2947; CST), p27 (#3686; CST), Cyclin E1 (#11935-1-AP; Proteintech), CDK2 (#10122-1-AP; Proteintech), phospho-CDK2 (#4539; CST), GLUT1(#73015; CST), PGK1(A12686; ABclonal), β-Tubulin (#10094-1-AP; Proteintech), Lamin B1 (#13435; CST), and β-Actin (#66009-1-Ig; Proteintech). All full-length, uncropped western blot images are provided in the Supplemental Material.

### Immunofluorescence

Cells grown on coverslips in 24-well plates were treated with the specified drugs for 24 h. After PBS washing, the cells were fixed with 4% paraformaldehyde (PFA) for 15 min at room temperature (RT), permeabilized with 0.3% Triton X-100/PBS (15 min, RT), and blocked with 5% BSA/PBS (1 h, RT). The cells were then incubated overnight at 4 °C with anti-FoxO1 primary antibody (#2880; CST) diluted in blocking buffer, washed with PBS, and incubated with fluorophore-conjugated secondary antibody (1 h, RT, in the dark). After additional PBS washes, actin filaments were labeled with phalloidin-iFluor 594 (30 min, RT), and nuclei were counterstained with DAPI (10 min, RT). Finally, imaging was performed using a fluorescence microscope.

### Plasmid construction

The sequence of the *FoxO1* promoter was obtained from the UCSC database (https://genome.ucsc.edu/) and compared and validated in NCBI. The binding site of PPARα in the *FoxO1* promoter region was predicted using the JASPAR database (https://jaspar.genereg.net/).

Human *FoxO1* promoter fragments were amplified by PCR from human genomic DNA with the primers 5′-GCTAGCTGCAGAATTACTTGTAAGAA-3′ and 5′-AAGCTTGCATGCGGCACCGCCGCCCG-3′. The PCR products were digested by *Nhe* I and *Hind* III and cloned into the reporter plasmid pGL3-Basic (Promega Corp., Madison, WI, USA) using T4 ligase to generate the pGL3-*FoxO1* promoter-LUC. Similarly, human PPARα (5′-GCGTGCACGGCACAACCAGCACCAT-3′ and 5′CGGGATCCTGCTCCCCCGTCTCCTTTG-3′) and human *FoxO1* (5′-AGATCTATGGCAGCCTCAGGGGT-3′ and 5′-GGTACCTCAGCGTCCTCTGGGTGG-3′) were cloned into the pCMV vector (Promega) to generate pCMV-3× FLAG-*PPARα* and pCMV-SPORT6-*FoxO1*, respectively.

### Transfection and luciferase reporter assay

pGL3-*FoxO1* promoter-LUC, PML-SV40-HRLUC, and pCMV-3×FLAG-PPARα were co-transfected into HEK293T cells via Lipofectamine 2000 (Thermo Fisher Scientific, Waltham, MA, USA) for 36 h. The cells were then washed with pre-cooled 1× PBS and lysed with Harvest Buffer (200 μL/per well) for 10 min. After centrifugation, the supernatant was mixed with the luciferase substrate, and the luminescence intensity was measured within 30 s. Firefly luciferase activity values were divided by Renilla luciferase activity values to obtain the normalized luciferase activities (Promega).

### Chromatin immunoprecipitation assay

Briefly, chromatin immunoprecipitation (ChIP) was performed to examine PPARα recruitment at the *FoxO1* promoter region using the SimpleChIP Enzyme-Assisted Chromatin Immunoprecipitation Kit (#9003, Cell Signaling Technology). Chromatin was immunoprecipitated using an anti-PPARα antibody (#ab227074, Abcam), and the ChIP- DNA was subsequently quantified via quantitative real-time PCR using specific *FoxO1* promoter primers (5’-tcggccccctgccctgcca-3’ and 5’-ctacggagcagcagagccg-3’).

### Immunohistochemistry

Briefly, formalin-fixed, paraffin-embedded tissue sections were deparaffinized in xylene and rehydrated through a graded ethanol series. For IHC, antigen retrieval was performed in citrate buffer (pH 6.0) using a microwave heating method. Endogenous peroxidase was blocked with 3% H_2_O_2_, and the slides were subsequently incubated with anti-Ki67 primary antibody (A20018; ABclonal) overnight at 4 °C, followed by HRP-conjugated secondary antibody incubation. Diaminobenzidine (DAB) was used as the chromogen, and the sections were counterstained with hematoxylin. For H&E staining, the sections were stained with Mayer’s hematoxylin and eosin Y as per standard protocols.

### Statistical analysis

Analyses were performed in GraphPad Prism 10 (GraphPad Software, USA). Unless otherwise stated, all tests were two-sided. Data are presented as the mean ± standard deviation (SD), with *n* indicating independent biological replicates. In vitro experiments were conducted with *n* = 3 independent repeats; xenograft studies were conducted with *n* = 5 mice per group for CDX and *n* = 6 per group for PDX. For single-factor designs, one-way analysis of variance (ANOVA) was followed by Tukey’s (all pairwise) or Dunnett’s (vs. control) multiple comparisons; for two-factor designs and time-course data (e.g., tumor volume and body weight), two-way repeated-measures ANOVA was used. Normality and homogeneity of variance were assessed (Shapiro–Wilk; Brown–Forsythe). Drug synergy was quantified using the Chou–Talalay CI calculated in CompuSyn (CI < 1 indicates synergy). For omics-level analyses (RNA-seq, metabolomics, and gene set enrichment analysis/Kyoto Encyclopedia of Genes and Genomes (GSEA/KEGG)), multiple testing was controlled by the Benjamini–Hochberg false discovery rate (FDR); enrichment results are reported as normalized enrichment scores (NESs) with corresponding FDR q values. Statistical significance was set at *p* < 0.05 (or FDR *q* < 0.05, where applicable).

## Results

### Linperlisib combined with chiglitazar inhibits the growth of FL cells in vitro and in vivo

**Fig. 1:**
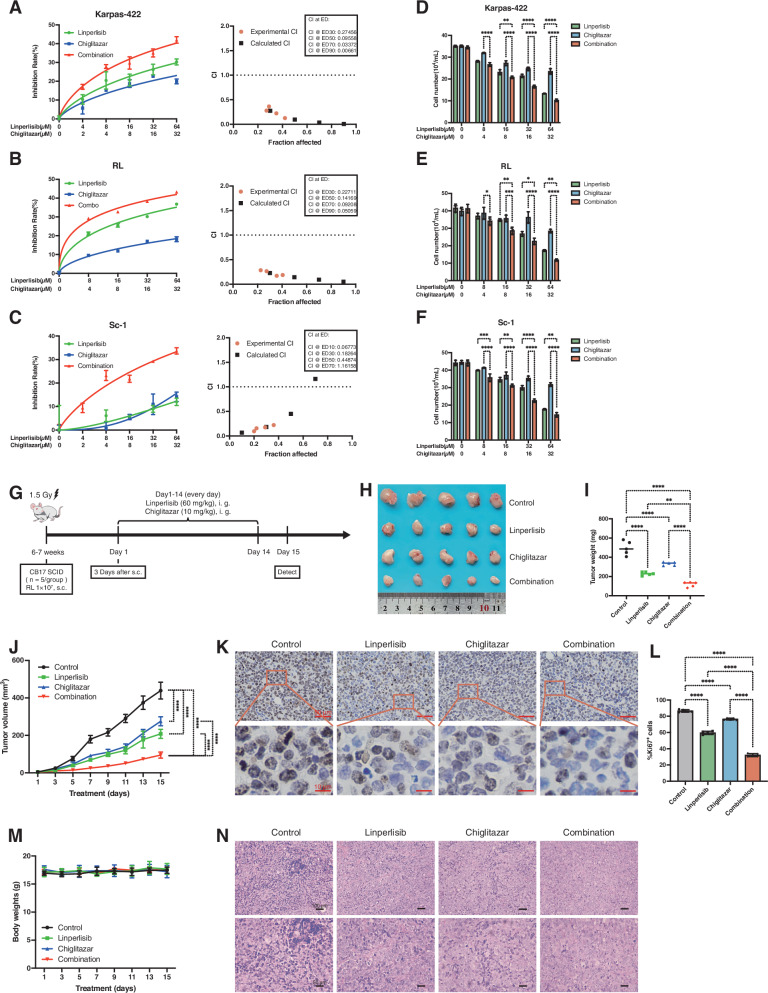
Linperlisib combined with Chiglitazar inhibits FL cell growth in vitro and in vivo. **A**–**C** CCK-8 viability and combination index (CI; Chou–Talalay) curves for Karpas-422, RL, and Sc-1 cells treated with linperlisib, chiglitazar, or both in combination; CI < 1 indicates synergy (*n* = 3 independent experiments). **D**–**F** Viable cell counts by trypan-blue exclusion in the same cell lines (*n* = 3). **G** CDX study design: RL cells (1 × 10^7^) were subcutaneously injected into CB17 SCID mice; linperlisib (60 mg/kg) and chiglitazar (10 mg/kg) were administered from day 3 for 14 days. **H** Representative tumors at endpoint. **I** Tumor weights at endpoint (*n* = 5 per group). **J** Tumor volume trajectories during treatment (*n* = 5 per group). **K** Representative Ki-67 IHC. Scale bars as indicated. **L** Quantification of Ki-67–positive cells (*n* = 5 per group). **M** Mouse body-weight monitoring over the study period (*n* = 5 per group). **N** H&E staining of tumor sections. Scale bars as indicated.

To evaluate the therapeutic potential of linperlisib and chiglitazar in FL, we conducted in vitro cell viability assays and in vivo xenograft studies. CCK-8 assays showed that treatment with either linperlisib or chiglitazar alone significantly reduced the viability of Karpas-422, RL, and Sc-1 cells; furthermore, the combination treatment resulted in a more pronounced inhibitory effect (Fig. 1A–C). Trypan blue staining confirmed the presence of significantly fewer viable cells in the combination group compared with that observed with monotherapy (Fig. 1D–F). The CI analysis revealed synergistic effects between the two drugs across all three FL cell lines.

In the RL CDX model (Fig. 1G), CB17-SCID mice (*n* = 5 per group) treated with the combination showed significantly reduced tumor burden compared to those receiving the control or monotherapy, with concordant decreases in tumor volume and endpoint weight (Fig. 1H–J). Immunohistochemical analysis demonstrated reduced Ki-67 expression in the combination group (Fig. 1K), and the statistical analysis confirmed a significantly lower Ki-67 positivity rate (Fig. 1L). No significant differences in body weight were observed among groups throughout the experiment, indicating good treatment tolerability (Fig. 1M). H&E staining revealed fewer mitotic figures and more necrotic regions in the combination group, further indicating its enhanced antitumor efficacy (Fig. 1N).

### Dual treatment with linperlisib and chiglitazar promotes apoptotic signaling in FL

**Fig. 2:**
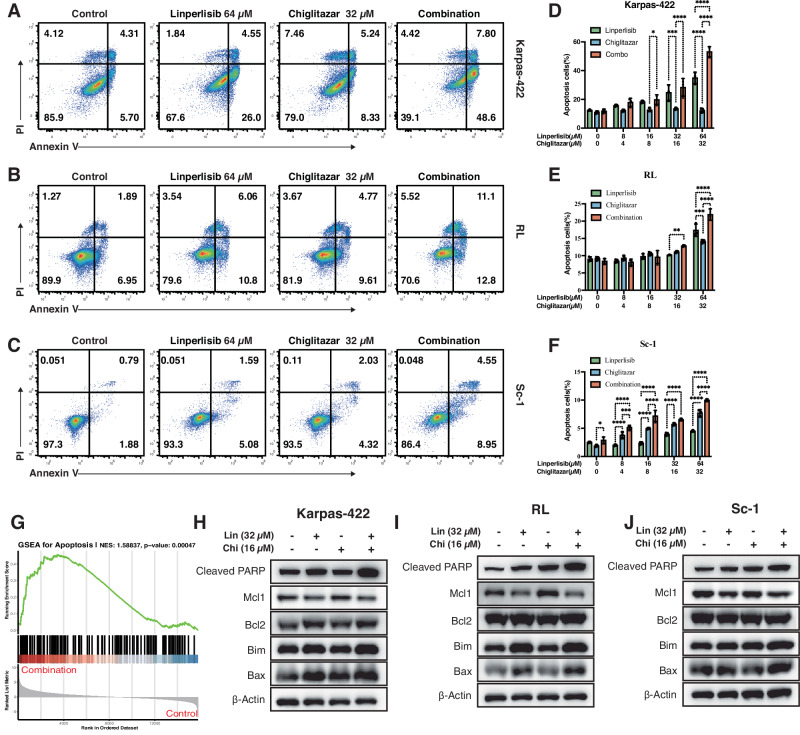
Linperlisib combined with Chiglitazar promotes apoptosis in follicular lymphoma cells. **A**–**C** Representative Annexin V/PI flow-cytometry plots in Karpas-422, RL, and Sc-1 cells after treatment. **D**–**F** Quantification of Annexin V–positive cells (*n* = 3). **G** Gene Set Enrichment Analysis (GSEA) showing enrichment of apoptosis-related pathways (NES = 1.58837, *p* = 0.00047). **H**–**J** Western blots of apoptosis-related proteins (cleaved PARP, Bcl-2, Mcl-1, Bim, Bax) in Karpas-422, RL, and Sc-1 cells; combination treatment increased cleaved PARP, Bim, and Bax and reduced Mcl-1, while Bcl-2 showed no consistent change (representative of three independent experiments).

Annexin V/PI double-staining flow cytometry was performed to determine whether the enhanced antitumor activity of linperlisib combined with chiglitazar was mediated by the induction of apoptosis in FL cells. In Karpas-422, RL, and Sc-1 cells, both monotherapies increased apoptosis, while the combination treatment resulted in a significantly higher apoptotic rate (Fig. 2A–C). Quantitative analysis confirmed this synergistic pro-apoptotic effect (Fig. 2D–F). GSEA further validated these findings, revealing significant enrichment of apoptosis-related gene sets in cells treated with the combination (NES = 1.58837, *p* = 0.00047; Fig. 2G).

To explore the underlying molecular mechanisms, we analyzed the expression of key apoptosis-related proteins through western blotting. The dual treatment consistently increased the levels of cleaved PARP, indicating activation of the intrinsic apoptotic pathway. Pro-apoptotic proteins such as Bim and Bax were also notably upregulated, particularly Bim, which showed robust elevation across all three cell lines (Fig. 2H–J). Anti-apoptotic protein expression, including that of Mcl-1, was downregulated following combination treatment, whereas Bcl-2 levels showed no consistent trend. These results suggest that linperlisib and chiglitazar synergistically promote apoptosis by modulating the expression of Bcl-2 family proteins and activating caspase-related signaling.

### Linperlisib and chiglitazar synergistically induce G1/S phase arrest in FL cells via regulation of cell cycle checkpoint proteins

**Fig. 3:**
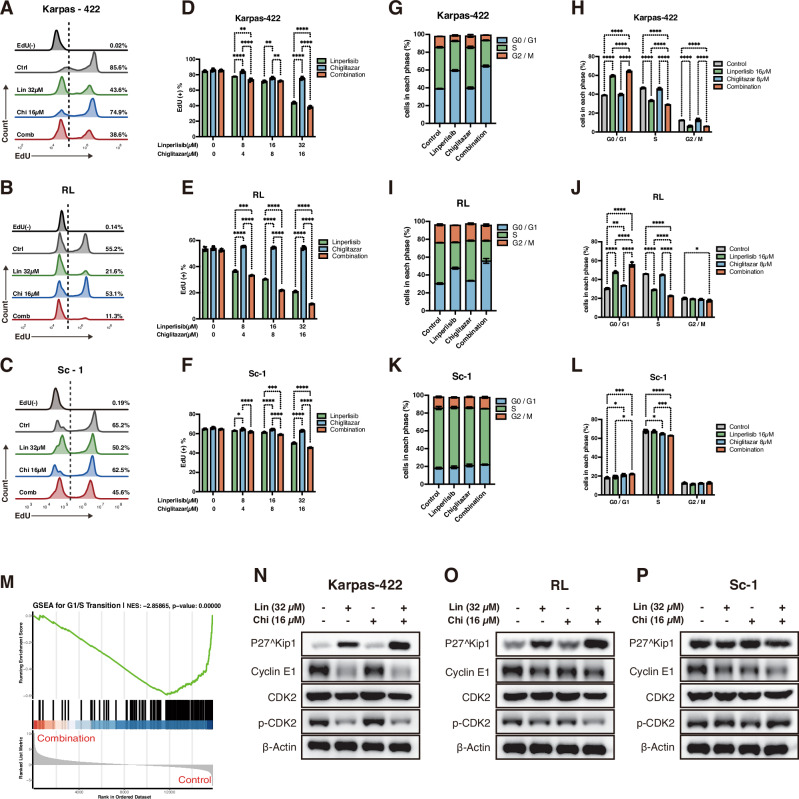
Linperlisib combined with Chiglitazar induces G1/S cell-cycle arrest. **A**-**C** EdU flow plots for Karpas-422, RL, and Sc-1 cells after 24-h treatment (Linperlisib 32 μM; Chiglitazar 16 μM). **D**–**F** Quantification of EdU-positive cells showing reduced DNA synthesis with the combination (*n* = 3). **G**–**L** (**G**, **H**) for Karpas-422; (**I**, **J**) for RL; (**K**, **L**) for Sc-1. Cell-cycle distribution by PI staining, demonstrating G0/G1 accumulation with Linperlisib and further enhancement by the combination (Linperlisib 16 μM; Chiglitazar 8 μM; *n* = 3). **M** GSEA indicating negative enrichment of the G1/S transition pathway (NES = − 2.85865, *p* < 0.00001). **N**–**P** Western blots of G1/S checkpoint proteins (p27, Cyclin E1, CDK2, p-CDK2); combination treatment increased p27 and decreased Cyclin E1, CDK2, and p-CDK2 (representative of three independent experiments).

EdU incorporation and cell cycle distribution assays were performed to determine whether linperlisib and chiglitazar suppress FL cell proliferation through cell cycle regulation. In Karpas-422, RL, and Sc-1 cells, combination treatment markedly reduced the proportion of EdU-positive cells, indicating suppression of DNA synthesis (Fig. 3A–C). Quantitative analysis confirmed a significant reduction in EdU incorporation compared to monotherapy or control conditions (Fig. 3D–F). Flow cytometric analysis of cell cycle distribution revealed an increase in the G0/G1 population, especially in the combination group, indicating pronounced G1/S-phase arrest (Fig. 3G–L). Supporting this, GSEA showed strong negative enrichment of genes involved in G1/S transition (NES = −2.85865, *p* = 0.00000; Fig. 3M).

To explore the molecular basis of this arrest, we examined key cell cycle checkpoint proteins by western blotting. In all three FL cell lines, co-treatment with linperlisib and chiglitazar led to CDK inhibitor p27 upregulation and Cyclin E1, CDK2, and phosphorylated CDK2 (p-CDK2) downregulation, indicating the effective disruption of the CDK2–Cyclin E1 complexes required for G1/S progression (Fig. 3N–P). These results collectively demonstrate that the dual treatment induces robust G1/S cell cycle blockade through the coordinated modulation of key regulatory proteins.

### Transcriptomic profiling identifies FoxO1 as a potential key effector mediating the synergistic response to linperlisib and chiglitazar

**Fig. 4:**
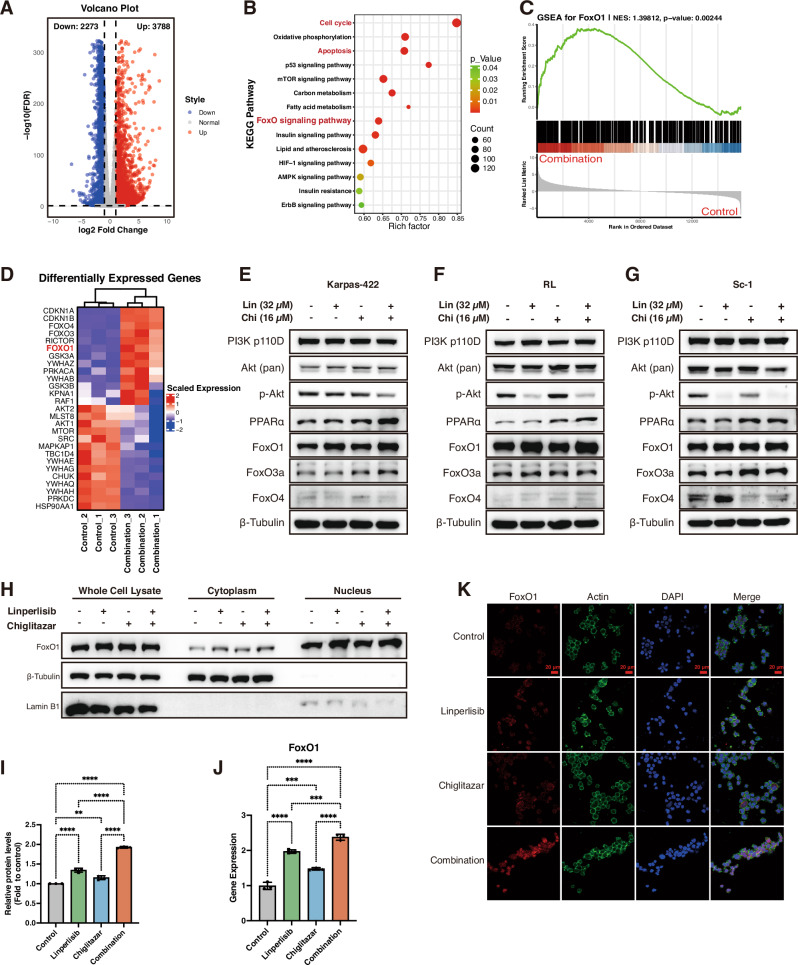
Transcriptomic and molecular profiling identify FoxO1 as a central effector of combination therapy. **A** Volcano plot of differentially expressed genes after combination treatment. **B** KEGG pathway enrichment highlighting apoptosis, cell-cycle, and FoxO signaling pathways. **C** GSEA showing enrichment of a FoxO1-related gene set (NES = 1.39812, *p* = 0.00244). **D** Heatmap of genes within the PI3K pathway (e.g., *CDKN1A*, *CDKN1B*, *FoxO1*, *FoxO3*, *FoxO4*). **E**–**G** Western blots for PI3K-p110δ, AKT, p-AKT, PPARα, and FoxO family proteins in Karpas-422, RL, and Sc-1 cells; the combination suppressed PI3K/AKT signaling and increased PPARα and FoxO1 (representative of three independent experiments). **H** Subcellular fractionation with Western blot showing increased nuclear FoxO1 after combination treatment (representative of three independent experiments). **I** Densitometric analysis of the nuclear/cytoplasmic ratio of FoxO1 (*n* = 3). **J** qPCR showing increased *FoxO1* mRNA after dual treatment (*n* = 3). **K** Immunofluorescence images demonstrating increased FoxO1 expression and nuclear localization following combination treatment. Scale bars as indicated.

To uncover the molecular mechanisms underlying the synergistic effects of linperlisib and chiglitazar, we performed transcriptomic profiling of FL cells treated with the combination regimen. Volcano plot analysis revealed a broad spectrum of differentially expressed genes, including substantial upregulation and downregulation events (Fig. 4A). KEGG pathway enrichment analysis identified multiple key pathways, notably those related to cell cycle regulation, apoptosis, and the FoxO signaling pathway (Fig. 4B). GSEA further confirmed significant enrichment of FoxO1-associated transcriptional programs (NES = 1.39812, *p* = 0.00244; Fig. 4C). Within the PI3K signaling network, genes including *CDKN1A*, *CDKN1B*, *FoxO1*, *FoxO3*, and *FoxO4* were prominently altered (Fig. 4D).

Western blotting analysis showed that combination treatment downregulated upstream PI3K/AKT components (p110δ, pan-AKT, and phosphorylated AKT) and upregulated PPARα and FoxO1, whereas FoxO3 and FoxO4 levels changed only modestly (Fig. 4E–G), suggesting selective activation of FoxO1 in response to PI3K/PPARα modulation. Subcellular fractionation assays revealed a pronounced nuclear accumulation of FoxO1 protein upon combination treatment (Fig. 4H), with densitometric analysis confirming a significant increase in its nuclear-to-cytoplasmic ratio (Fig. 4I). Accordingly, qPCR showed strong induction of *FoxO1* mRNA, exceeding that of *FoxO3* or *FoxO4* (Fig. 4J). Immunofluorescence imaging corroborated both increased FoxO1 expression and its enhanced nuclear localization following combination group (Fig. 4K). Collectively, these data suggest that FoxO1 acts as a key transcriptional effector downstream of PI3K/PPARα signaling, mediating the enhanced apoptotic and anti-proliferative responses elicited by linperlisib and chiglitazar co-treatment.

### Metabolomic analysis reveals that chiglitazar activates FoxO1 through metabolic reprogramming and glycolysis inhibition

**Fig. 5:**
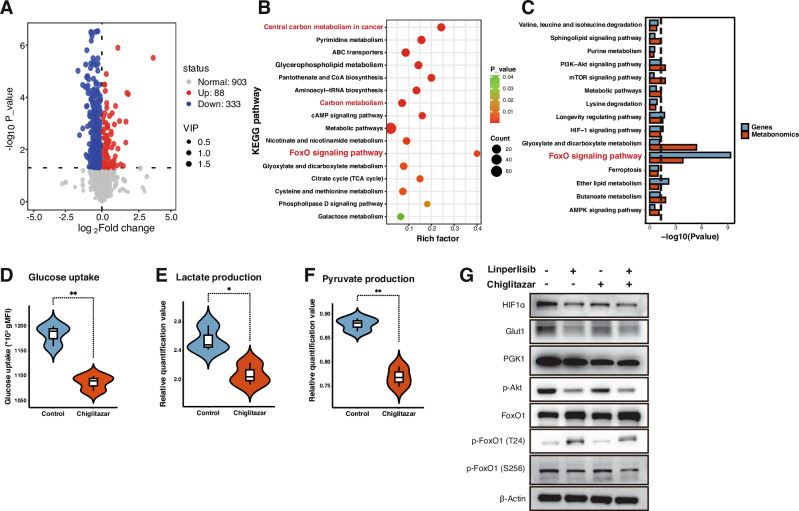
Chiglitazar reprograms cellular metabolism and promotes FoxO1 activation. **A** Volcano plot of differential metabolites in RL cells (Chiglitazar vs control: 88 upregulated, 333 downregulated). **B** KEGG enrichment of altered metabolites highlighting FoxO signaling, carbon metabolism, and central carbon metabolism in cancer. **C** Integrated KEGG analysis combining transcriptomic and metabolomic data, identifying the FoxO pathway among the top-enriched pathways. **D**–**F** Functional assays showing reduced glucose uptake, lactate production, and pyruvate levels after Chiglitazar (*n* = 3). **G** Western blots showing downregulation of HIF-1α, GLUT1, PGK1, p-AKT, and phosphorylated FoxO1 (T24, S256) (representative of three independent experiments).

Untargeted metabolomic profiling was performed in RL FL cells to characterize the effects of chiglitazar on FoxO1 activity. Volcano plot analysis revealed extensive metabolic remodeling, with a large number of significantly altered metabolites observed compared to that in the control (Fig. 5A). KEGG enrichment analysis of these differential metabolites highlighted the FoxO signaling pathway, carbon metabolism, and central carbon metabolism in cancer as among the most affected pathways (Fig. 5B).

An integrated KEGG analysis of combined metabolomic and transcriptomic datasets further identified the FoxO signaling pathway as a top-enriched node (Fig. 5C), suggesting a direct link between metabolic rewiring and transcriptional regulation. Functionally, chiglitazar significantly suppressed glycolytic flux, as evidenced by reduced glucose uptake (Fig. 5D), lactate production (Fig. 5E), and intracellular pyruvate leves (Fig. 5F).

Consistent with these metabolic changes, western blotting demonstrated decreased expression of key glycolytic regulators, including HIF1α, Glut1, and PGK1, along with reduced AKT phosphorylation. Notably, phosphorylation of FoxO1 at T24 and S256 was also suppressed, suggesting enhanced nuclear localization and transcriptional activity (Fig. 5G). In addition to these post-translational changes, *FoxO1* mRNA was upregulated by chiglitazar (Fig. 4J).

To determine whether this transcriptional activation was directly mediated by PPARα. we performed dual-luciferase reporter assays, which confirmed that PPARα activation robustly enhanced *FoxO1* promoter activity (Fig. S1A). Bioinformatic analysis predicted a PPARα response element (PPRE) at –402/–378 bp within the promoter (Fig. S1B). Mutation of this motif abolished chiglitazar-induced promoter activation in the luciferase assay (Fig. S1C), confirming its functional importance. ChIP-qPCR further demonstrated increased PPARα binding to the *FoxO1* promoter upon chiglitazar treatment (Fig. S1D). Together, these results indicate that chiglitazar activates FoxO1 transcriptionally via direct PPARα promoter engagement and post-translationally via glycolysis inhibition, thus contributing to its synergistic therapeutic effects with linperlisib.

### FoxO1 knockdown attenuates the synergistic cytotoxic effects of linperlisib and chiglitazar in FL cells

**Fig. 6:**
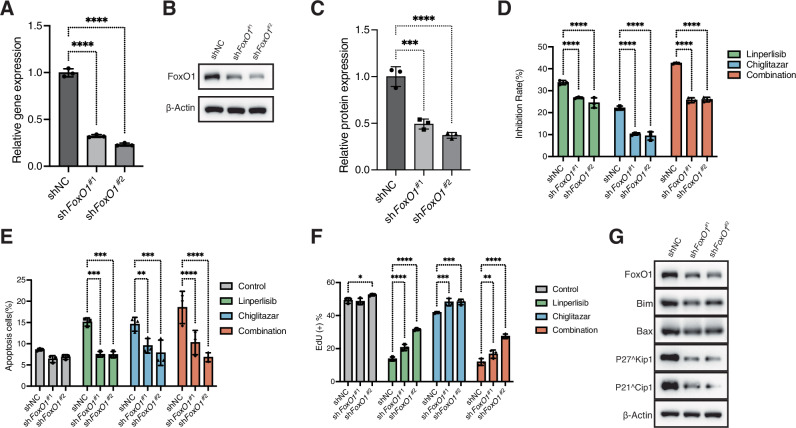
FoxO1 knockdown impairs the pro-apoptotic and anti-proliferative effects of the combination. **A** qPCR confirming *FoxO1* mRNA knockdown in RL cells stably expressing sh*FoxO1*#1 or sh*FoxO1*#2 (*n* = 3). **B** Western blot validation of FoxO1 protein reduction (representative of three independent experiments). **C** Densitometric analysis showing relative FoxO1 protein levels (~50% for sh*FoxO1*#1; ~37% for sh*FoxO1*#2; *n* = 3). **D** CCK-8 assays showing reduced drug sensitivity in FoxO1-knockdown cells treated with Linperlisib and Chiglitazar (*n* = 3). **E** Annexin V/PI apoptosis assays showing decreased apoptosis upon combination treatment in FoxO1-deficient cells (*n* = 3). **F** EdU assays showing increased DNA synthesis in knockdown cells under drug treatment (*n* = 3). **G** Western blots of FoxO1 downstream targets (Bim, Bax, p21, p27) in control and FoxO1-knockdown cells (representative of three independent experiments).

Two RL cell lines with stable FoxO1 knockdown (sh*FoxO1*#1 and sh*FoxO1*#2) were established to assess the essential role of FoxO1 in mediating the therapeutic synergy between linperlisib and chiglitazar. qPCR confirmed that *FoxO1* mRNA expression was reduced to approximately 32% and 24% of the control (shNC) levels, respectively (Fig. 6A). Consistently, western blotting revealed a marked decrease in FoxO1 protein levels (Fig. 6B), with densitometric analysis showing reductions to 50% and 37% of control levels (Fig. 6C).

Functional assays demonstrated that FoxO1 knockdown diminished the sensitivity of RL cells to the combination treatment. CCK-8 assays showed significantly higher viability in FoxO1-deficient cells following treatment with linperlisib and chiglitazar (Fig. 6D). Flow cytometric analysis using Annexin V/PI staining revealed decreased apoptosis rates in the knockdown cells upon drug treatment compared with those observed in the controls (Fig. 6E). In parallel, EdU incorporation assays showed increased proportions of proliferating cells, indicating that FoxO1 deficiency alleviated drug-induced replication stress (Fig. 6F).

At the molecular level, western blotting showed that FoxO1 knockdown reduced the expression of key pro-apoptotic proteins Bim and Bax, along with the CDK inhibitors p21 and p27 (Fig. 6G). These results collectively demonstrate that FoxO1 is required for the full induction of apoptosis and cell cycle arrest induced by linperlisib and chiglitazar; its knockdown attenuates the anti-proliferative and pro-apoptotic effects of combination therapy, underscoring its critical role as a downstream effector.

### Linperlisib and chiglitazar synergistically suppress tumor growth and activate FoxO1 signaling in an FL PDX model

**Fig. 7:**
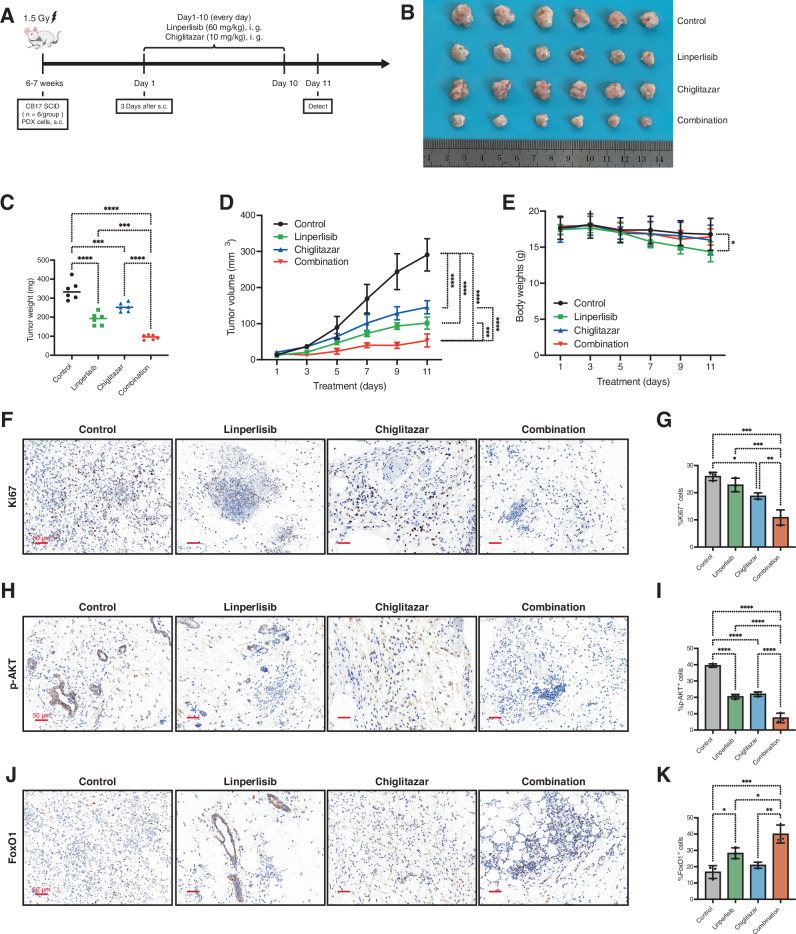
Linperlisib and Chiglitazar synergistically suppress tumor growth and activate FoxO1 signaling in an FL PDX model. **A** PDX study design. **B** Representative tumor images at endpoint. **C** Tumor weights at endpoint (*n* = 6 per group). **D** Tumor volume curves over time (*n* = 6 per group). **E** Mouse body-weight curves; a mild reduction was observed in the Linperlisib group (*n* = 6 per group). **F** Ki-67 IHC and **G** quantification (*n* = 6 per group). Scale bars as indicated. **H** p-AKT IHC and **I** quantification showing reduced PI3K pathway activation in the combination group (*n* = 6 per group). Scale bars as indicated. **J** FoxO1 IHC and **K** quantification showing enhanced nuclear FoxO1 in the combination group (*n* = 6 per group). Scale bars as indicated.

We established a PDX model of FL to evaluate the therapeutic efficacy of linperlisib combined with chiglitazar in a clinically relevant setting. Compared with the control and monotherapy groups, the combination group exhibited markedly reduced tumor volumes and weights, as confirmed by tumor resection images and measurements (Fig. 7A–C). Tumor growth curves further demonstrated that the combination treatment yielded the most significant inhibition of tumor progression (Fig. 7D). Mice in the linperlisib group showed mild body weight loss; however, this effect was not exacerbated by combination treatment (Fig. 7E). Immunohistochemical analysis revealed a significant reduction in tumor cell proliferation in the combination group, as shown by Ki67 staining and quantification (Fig. 7F, G). Furthermore, p-AKT expression was the lowest in the combination group, indicating more effective inhibition of PI3K/AKT signaling (Fig. 7H, I). In contrast, FoxO1 nuclear localization was the highest in the combination group, suggesting that PI3K inhibition and metabolic reprogramming synergistically restore FoxO1 activity in vivo (Fig. 7J, K). Taken together, these results establish the AKT–FoxO1 signaling pathway as a critical downstream target of the combination treatment in FL.

## Discussion

This study establishes that combined targeting of PI3Kδ and PPARα with linperlisib and chiglitazar produces synergistic antitumor activity in FL. We identify the reactivation of the transcription factor FoxO1 as the central integrator through which this combination concurrently suppresses oncogenic PI3K/AKT signaling and reprograms tumor metabolim, thereby overcoming the adaptive resistance that frequently limits PI3Kδ inhibitor monotherapy. Although PI3Kδ inhibition has demonstrated clinical activity in relapsed/refractory FL, treatment responses are often incomplete and curtailed by adaptive resistance [27–32]. Our work expands this understanding by revealing a previously unrecognized regulatory axis in which PPARα‑driven metabolic modulation reactivates FoxO1 tumor‑suppressive function through both transcriptional and post‑translational mechanisms. This highlights the mechanistic novelty and therapeutic potential of targeting metabolic-signaling convergence in lymphoid malignancies.

Specifically, our results demonstrate that chiglitazar markedly suppresses glycolysis, as evidenced by decreased glucose uptake and reductions in lactate and pyruvate production. These metabolic alterations are consistent with previous research indicating chiglitazar’s role in modulating glucose and lipid metabolism through PPAR activation [33–37]. Importantly, these changes were accompanied by increased nuclear localization and transcriptional activity of FoxO1, driven by PPARα-mediated upregulation and AKT-dependent phosphorylation attenuation [38]. To delineate the transcriptional mechanism more precisely, we identified a predicted PPARα response element (PPRE) at −402/–378 bp within the FoxO1 promoter, confirmed direct PPARα binding via ChIP‑qPCR, and demonstrated that mutating this element abolishes chiglitazar‑induced promoter activation (Fig. S1B–D). These findings establish a direct PPARα–FoxO1 transcriptional regulatory axis, demonstrating that chiglitazar activates FoxO1 not only through metabolic rewiring but also through direct promoter engagement [39].

Functional validation using FoxO1 knockdown confirmed FoxO1’s central role in mediating the effects of the combination therapy. Knockdown of FoxO1 significantly reduced drug-induced apoptosis and cell cycle arrest while restoring cell proliferation. Concurrently, the expression levels of key downstream targets, including Bim, Bax, p21, and p27, were markedly decreased. These results not only confirm the mechanistic indispensability of FoxO1 in driving the therapeutic response but also corroborate its established role as a tumor suppressor in B-cell malignancies, consistent with previous findings [40–43].

Our in vivo results align closely with the in vitro findings, demonstrating significant tumor growth inhibition by the combination therapy in both CDX and PDX FL models. The use of both models strengthens the translational relevance of our findings. In PDX tumors, immunohistochemistry demonstrated a marked increase in FoxO1 expression in the combination group (Fig. 7J, K), which accompanied the most pronounced reductions in tumor burden and Ki67 proliferation. Although nuclear localization was not directly assessed in vivo, the strong correlation between FoxO1 upregulation and treatment response supports its potential value as a pharmacodynamic biomarker [44]. These data not only substantiate FoxO1 activation as a mechanistic endpoint but also suggest its potential as a pharmacodynamic biomarker to monitor treatment response in vivo. Notably, the combination therapy did not cause additional adverse effects in comparison with linperlisib monotherapy, such as weight loss, consistent with clinical reports of linperlisib’s tolerability. These findings further support the clinical potential of the combination therapy.

The rationale for selecting linperlisib and chiglitazar was based on their distinct yet complementary mechanisms of action. Linperlisib was chosen for its high PI3Kδ selectivity and improved safety relative to earlier PI3K inhibitors such as idelalisib, whereas chiglitazar was selected for its ability to remodel tumor metabolism at clinically relevant concentrations [12, 14, 45]. Together, these agents form a multilayered therapeutic strategy that simultaneously targets oncogenic signaling and metabolic reprogramming. Chiglitazar, a pan-PPAR agonist, has demonstrated anti-tumor effects in multiple contexts; however, its efficacy in FL is limited when used alone [46]. Our dose selection was guided by prior pharmacologic studies in hematologic models, and we hypothesized that metabolic modulation through PPAR activation could synergize with PI3Kδ inhibition to overcome adaptive resistance. Additionally, linperlisib was selected for its favorable pharmacodynamic properties and clinical safety profile, including its selectivity for PI3Kδ and reduced off-target toxicities compared to earlier agents such as idelalisib.

Regarding clinical application, we envision this combination therapy as a promising strategy for relapsed or refractory FL, particularly in patients who exhibit resistance to PI3K monotherapy. Given its favorable tolerability and dual mechanistic action, this regimen could also be explored as a frontline option in biomarker‑defined subgroups, such as FoxO1‑low or glycolysis‑high FL. Incorporating FoxO1 expression into future clinical trial designs may facilitate patient stratification and enhance therapeutic precision [47].

Despite these promising findings, several limitations exist. First, the number of patient-derived models used is limited; thus, we might not have fully captured the biological heterogeneity observed among FL patients. Expanding the number and diversity of patient-derived models in future studies will be crucial for enhancing the generalizability of these findings. In addition, this study did not explore the long-term safety or potential resistance mechanisms in detail; these aspects should be explored in subsequent research. Furthermore, the comprehensive effects of this combination therapy on the tumor microenvironment and immune system warrant systematic evaluation in future experiments. These limitations notwithstanding, our study lays a solid foundation for the preclinical rationale and clinical translation of this therapeutic approach.

This study’s findings have significant implications for cancer research and clinical practice. Given our identification of FoxO1 as a critical mediator linking metabolism and transcriptional regulation, we propose a novel combinational therapeutic strategy using linperlisib and chiglitazar and provide both theoretical and practical insights into overcoming resistance mechanisms in FL treatment. This combination treatment approach could potentially enhance therapeutic outcomes in FL patients and provide a valuable framework for addressing treatment challenges in other B-cell lymphomas.

## Supplementary information

SUPPLEMENTAL MATERIAL

Full and uncropped western blots

## Data Availability

The RNA-seq data have been deposited in the Gene Expression Omnibus (GEO) under accession number GSE311442. All other datasets presented in this study are available upon reasonable request to the corresponding author. The data may be obtained from the corresponding authors on reasonable request.
