# Supplementary material for: Dual targeting of PI3Kδ and PPARα enhances antitumor activity via FoxO1 activation in follicular lymphoma
Source: Cell Death Dis. 2026 Mar 23;17(1):341. doi: 10.1038/s41419-026-08593-5 (PMC13040022; doi:10.1038/s41419-026-08593-5)
Supplement: Supplementary file 1 — SUPPLEMENTAL MATERIAL [file 41419_2026_8593_MOESM1_ESM.pdf]

Dual Targeting of PI3Kδ and PPARα Enhances Anti-Tumor Activity via FoxO1 Activation in Follicular Lymphoma

Supplementary materials

Table S1

Name	Application	Sequence 5' to 3'
Foxo1-F	qPCR	CTCACCCTGTCGCAGATCTACGAG
Foxo1-R	qPCR	GTTGTCCATGGACGCAGCTCTTCTC
Actin-F	qPCR	CATGTACGTTGCTATCCAGGC
Actin-R	qPCR	CTCCTTAATGTCACGCACGAT
sh*FoxO1*#1	Knockdown	tgggagactctcacccattat
sh*FoxO1*#2	Knockdown	cagcaatgatgactttgataa

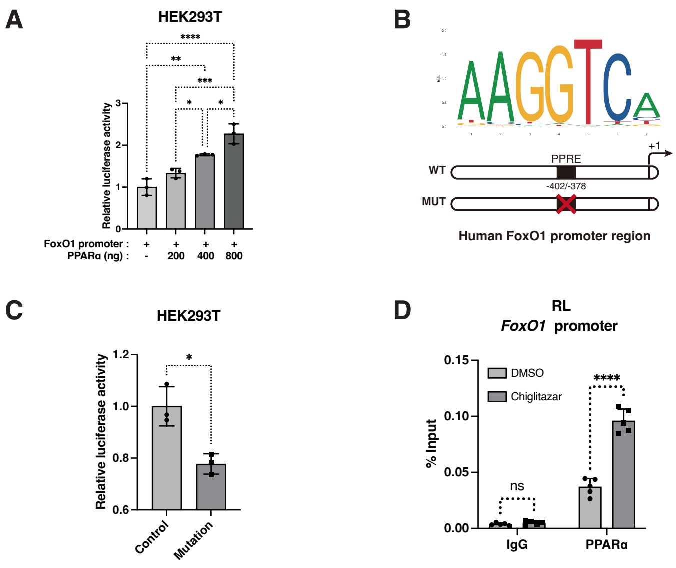

Figure S1. PPARα directly regulates the FOXO1 promoter.
(A) Dose-dependent activation of the FOXO1 promoter by chiglitazar measured via dual-luciferase assay (n = 3).
(B) Schematic of the FOXO1 promoter indicating a predicted PPARα response element (PPRE, "AAGGTCA") at –402/–378 bp upstream of the transcription start site.

(C) Luciferase assay comparing wild-type (WT) and PPRE-mutant (MUT) FOXO1 promoters; mutation of the PPRE abolishes chiglitazar-induced promoter activation (n = 3).

(D) ChIP-qPCR demonstrating enrichment of PPARα binding at the –402/–378 bp region of the FOXO1 promoter after chiglitazar treatment (n = 5).
