# Supplementary material for: Dual targeting of PI3Kδ and PPARα enhances antitumor activity via FoxO1 activation in follicular lymphoma
Source: Cell Death Dis. 2026 Mar 23;17(1):341. doi: 10.1038/s41419-026-08593-5 (PMC13040022; doi:10.1038/s41419-026-08593-5)
Supplement: Supplementary file 2 — Full and uncropped western blots [file 41419_2026_8593_MOESM2_ESM.pdf]

qPCR

	Ctrl	Lin	Chi	Comb
Actin	17.6631156	17.7572752	17.9712189	17.6130018
	17.4287343	17.9712189	17.4289305	17.5725093
	17.2823178	17.4289305	17.6631156	17.7276957
FoxO1	24.4793088	23.6456821	24.1218052	23.595017
	24.4226821	23.5231324	24.0352134	23.2581542
	24.2939164	23.5923356	24.0485904	23.3548198

ChIP-qPCR

Input		IgG		PPRE-PPARa	
24.85	24.39	28.32	29.06	25.30	23.74
24.56	24.01	28.80	27.34	25.23	23.20
29.63	29.72	33.49	33.83	30.07	28.87
30.00	30.24	34.66	33.36	30.87	29.70
29.08	29.48	33.21	33.06	30.26	28.98

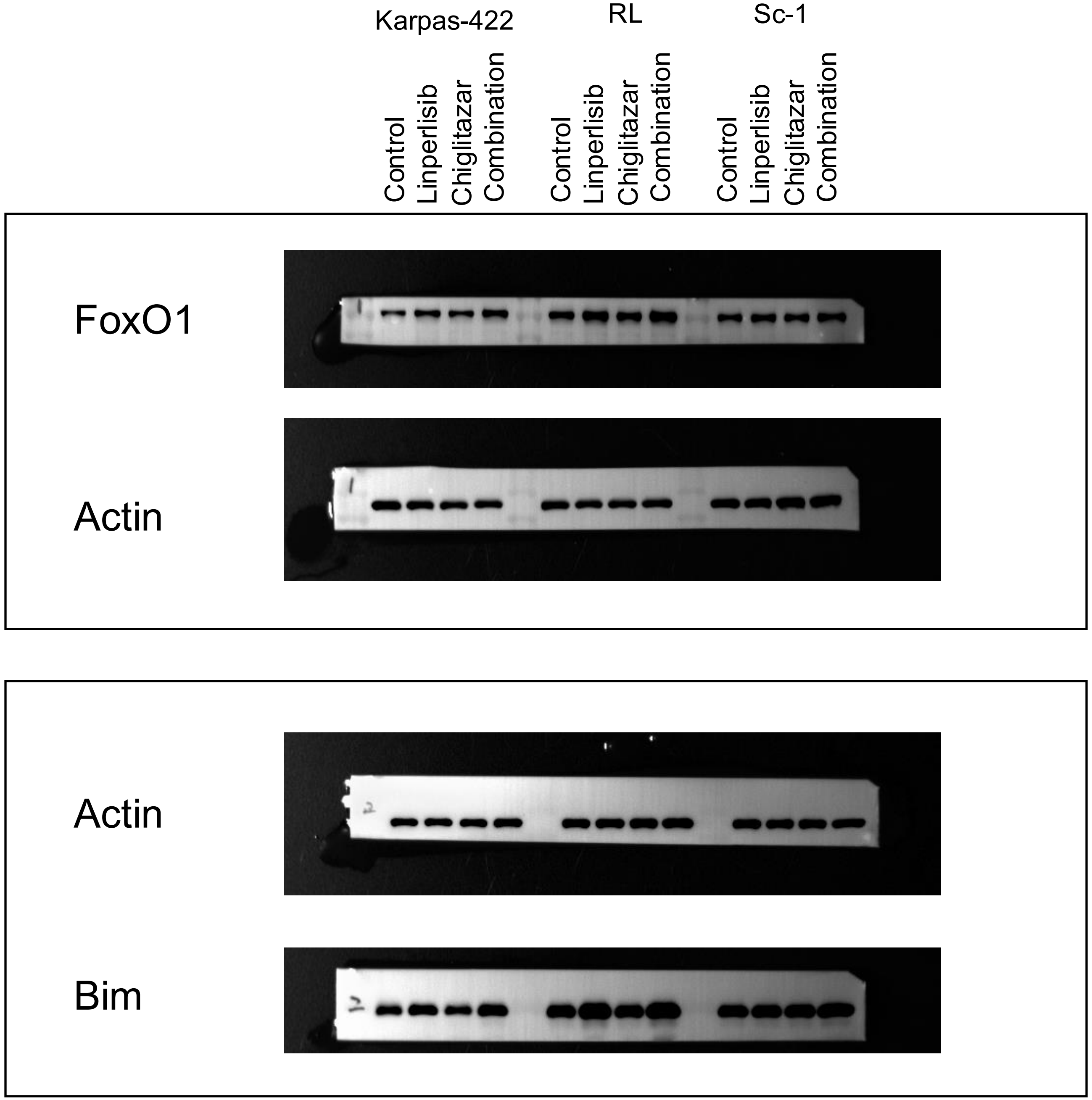
Karpas-422
RL
Sc-1
Control
Linperlisib
Chiglitazar
Combination
Control
Linperlisib
Chiglitazar
Combination
Control
Linperlisib
Chiglitazar
Combination
FoxO1
Actin
Actin
Bim

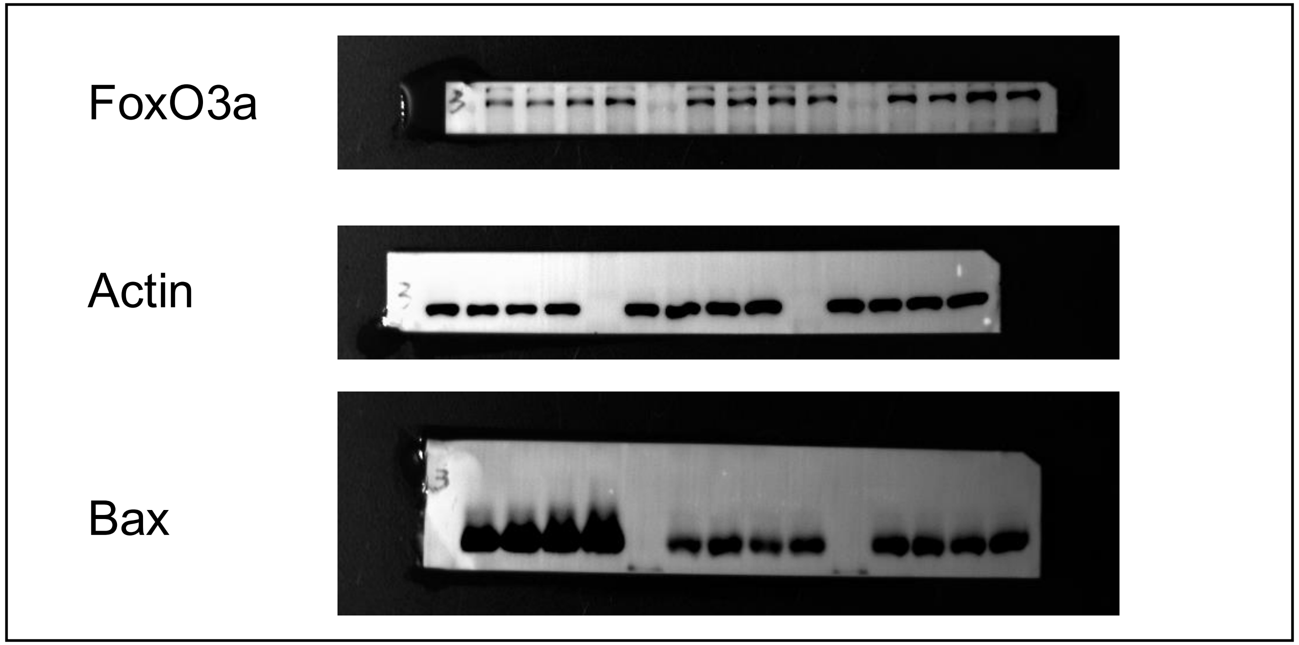
FoxO3a
Actin
Bax

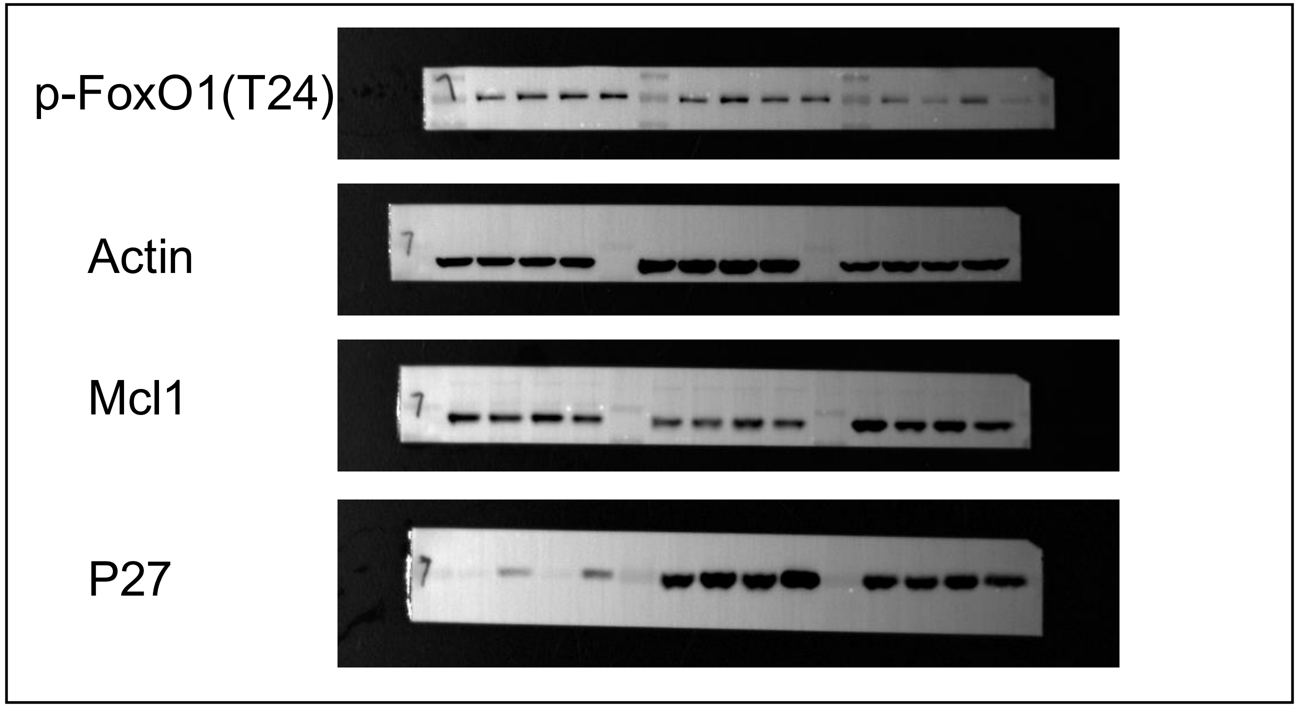
p-FoxO1(T24)
Actin
Mcl1
P27

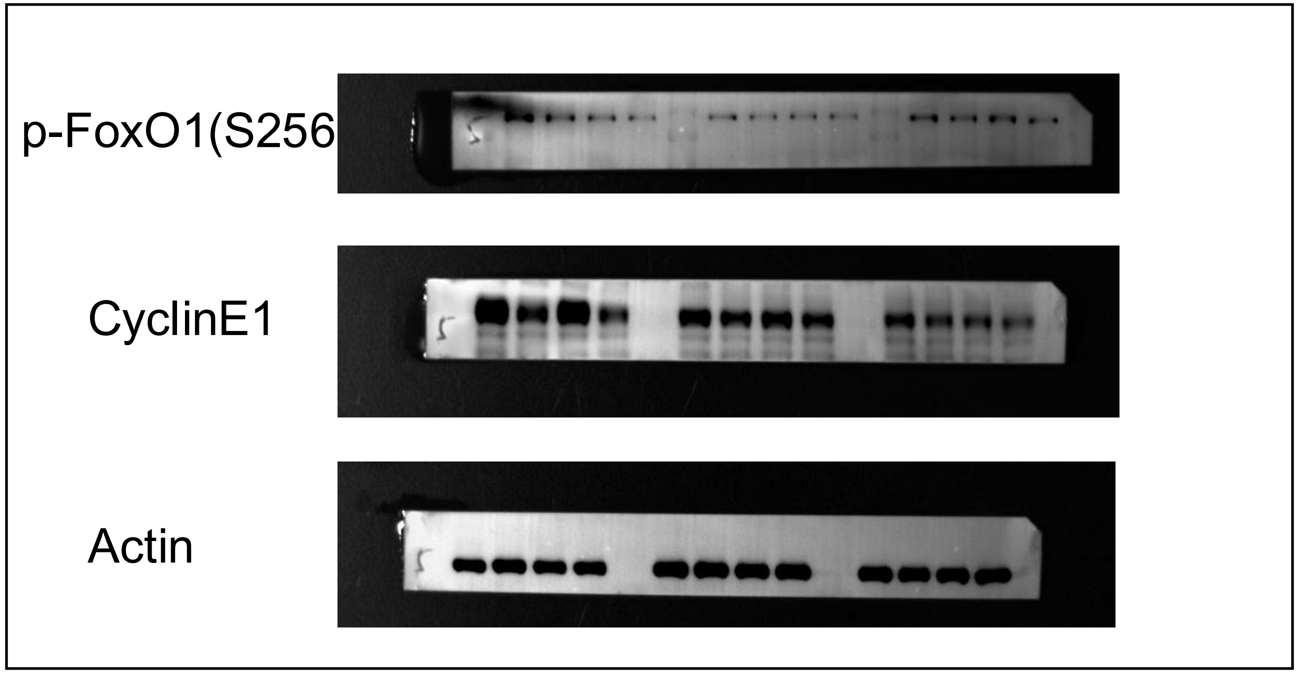
p-FoxO1(S256
CyclinE1
Actin

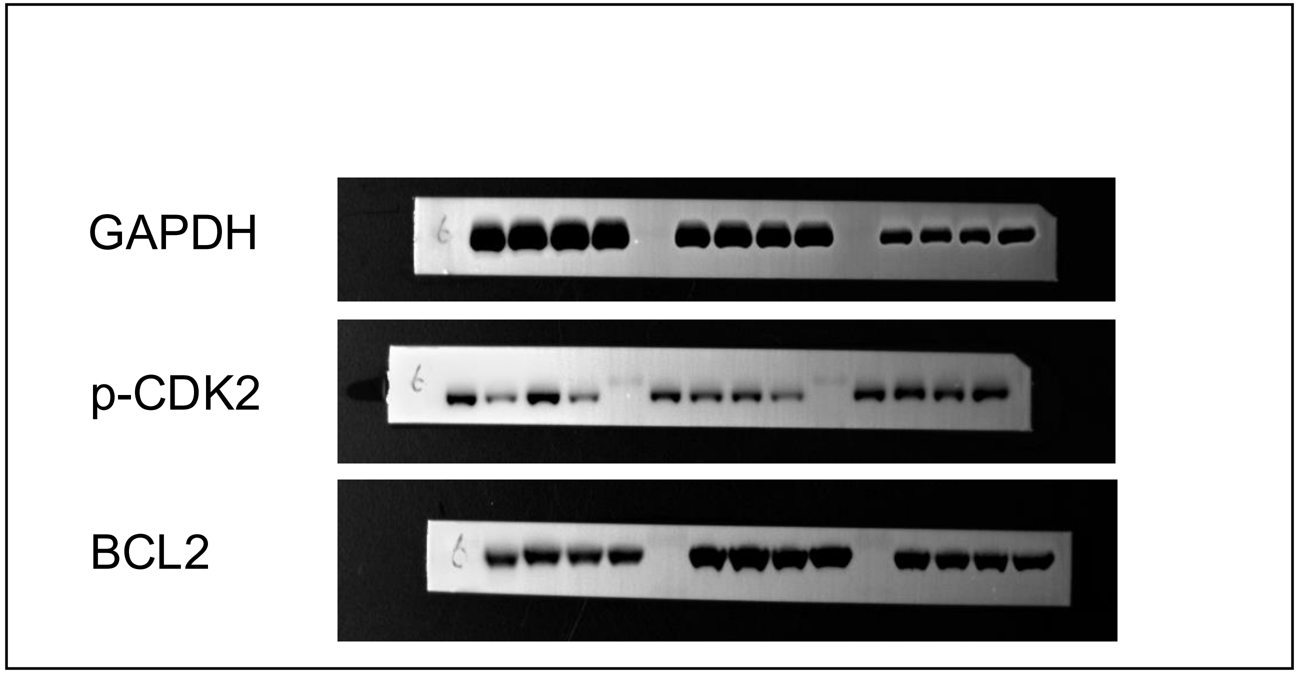
GAPDH
p-CDK2
BCL2

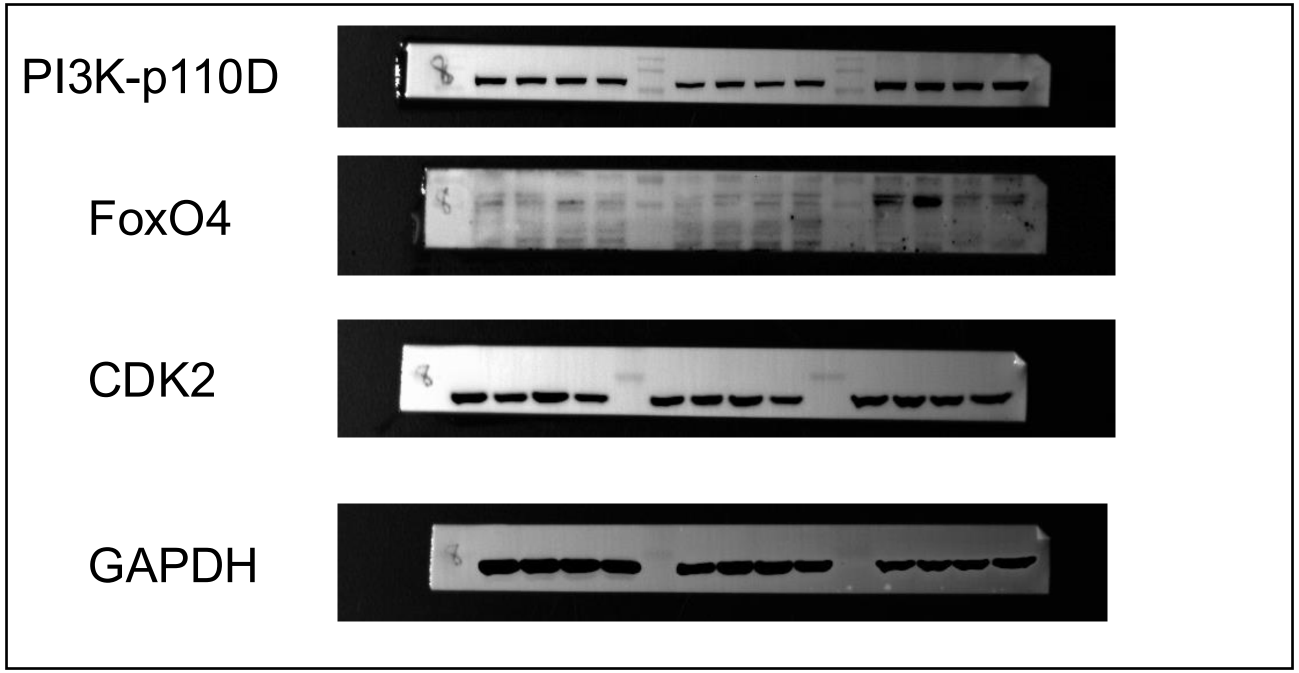
PI3K-p110D
FoxO4
CDK2
GAPDH

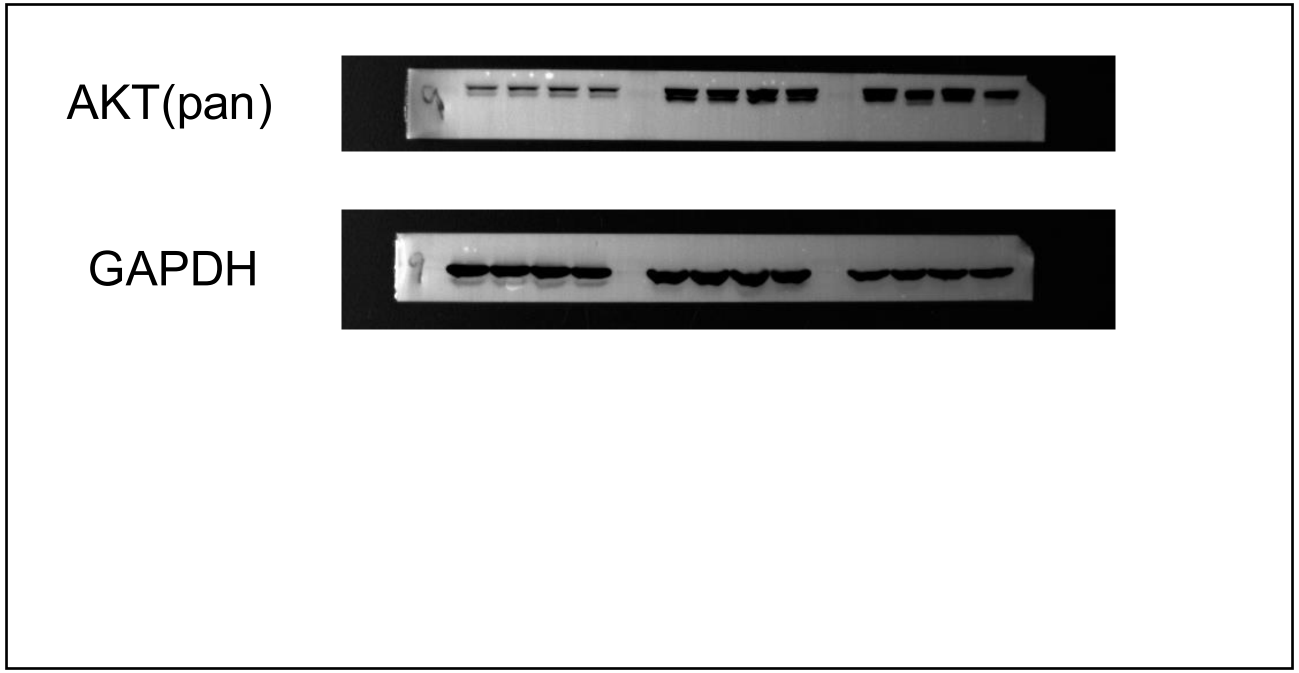
AKT(pan)
GAPDH

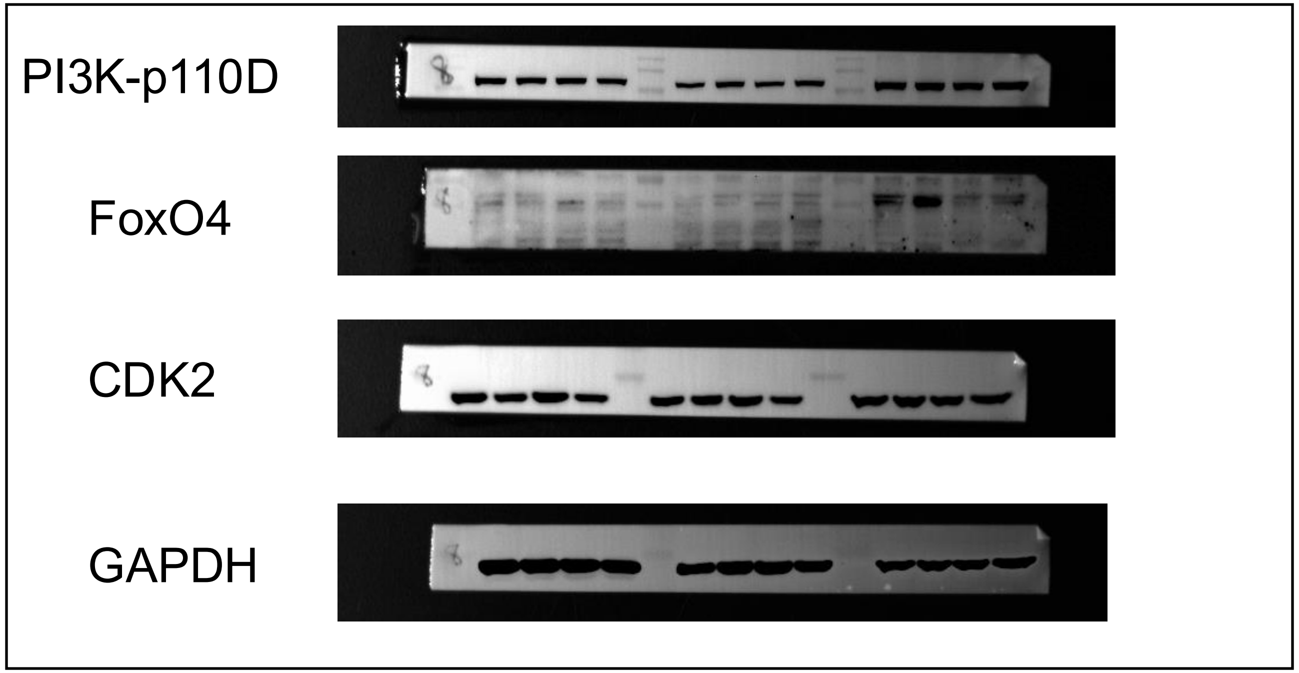
PI3K-p110D
FoxO4
CDK2
GAPDH

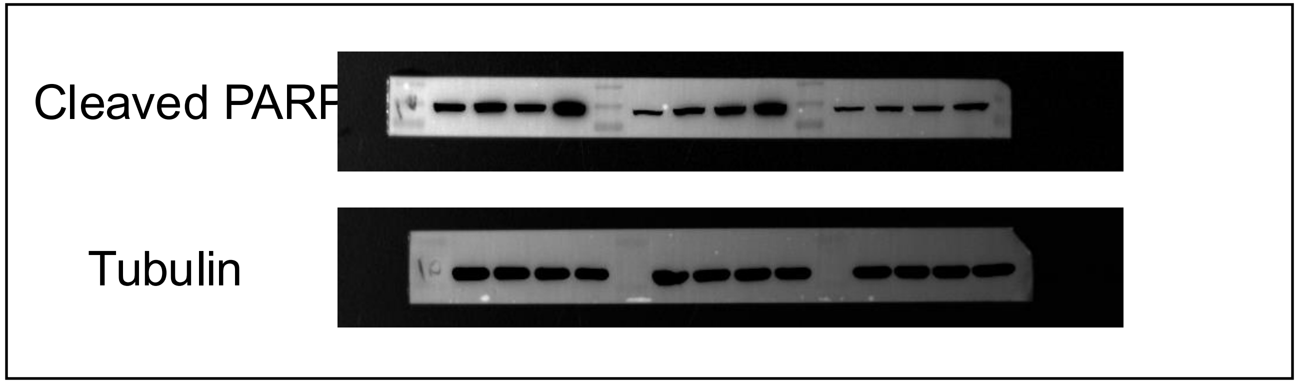
Cleaved PAR
Tubulin

RL

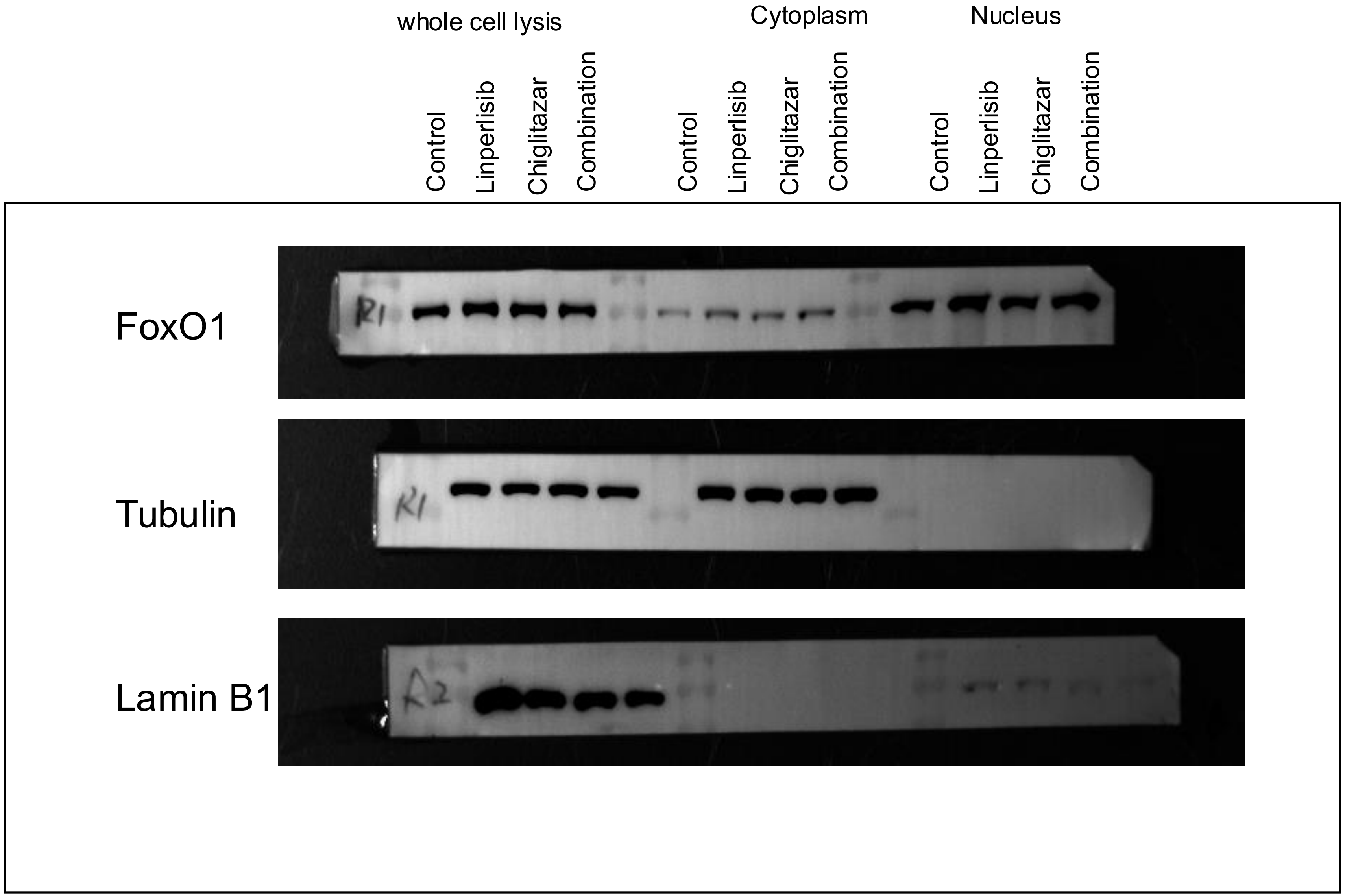
whole cell lysis
Cytoplasm
Nucleus
Control
Linperlisib
Chiglitazar
Combination
Control
Linperlisib
Chiglitazar
Combination
Control
Linperlisib
Chiglitazar
Combination
FoxO1
Tubulin
Lamin B1

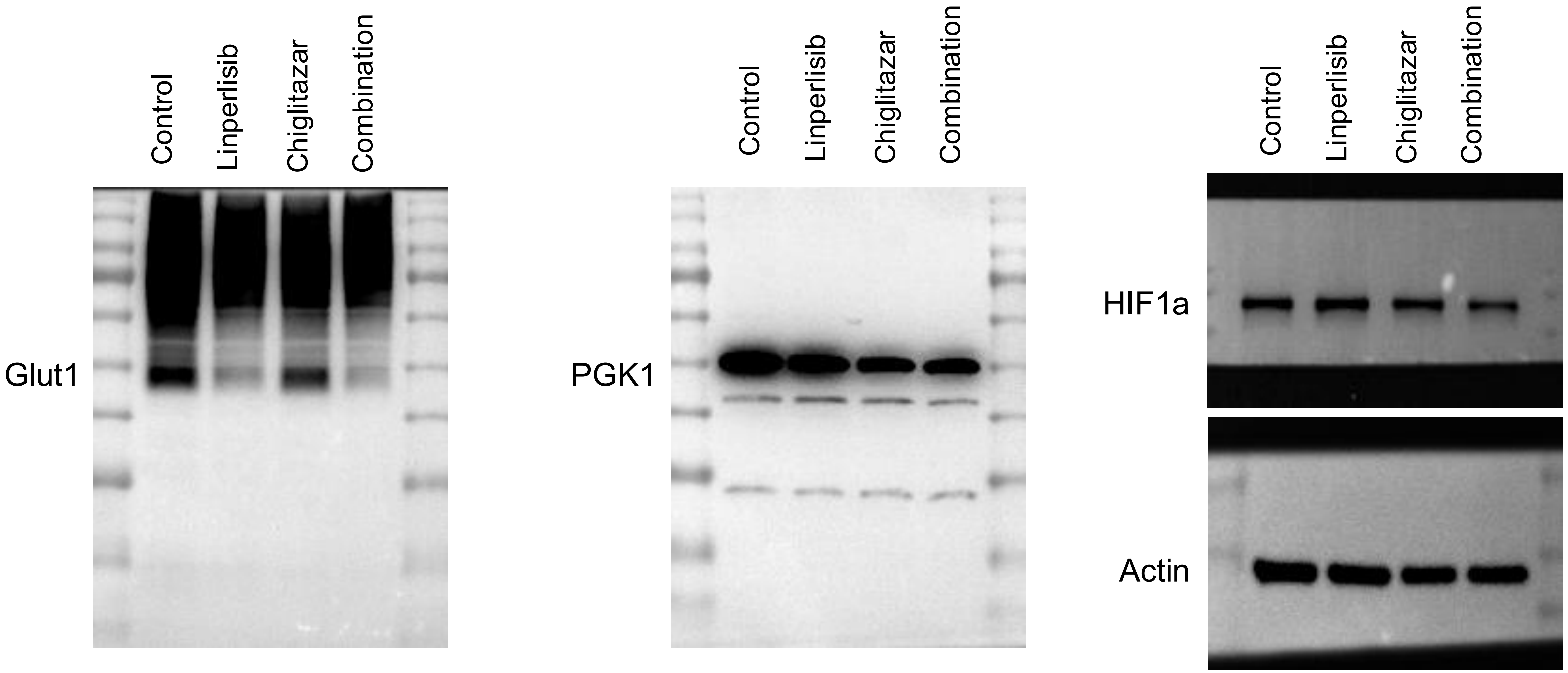
Control
Linperlisib
Chiglitazar
Combination
Glut1
Control
Linperlisib
Chiglitazar
Combination
PGK1
Control
Linperlisib
Chiglitazar
Combination
HIF1a
Actin

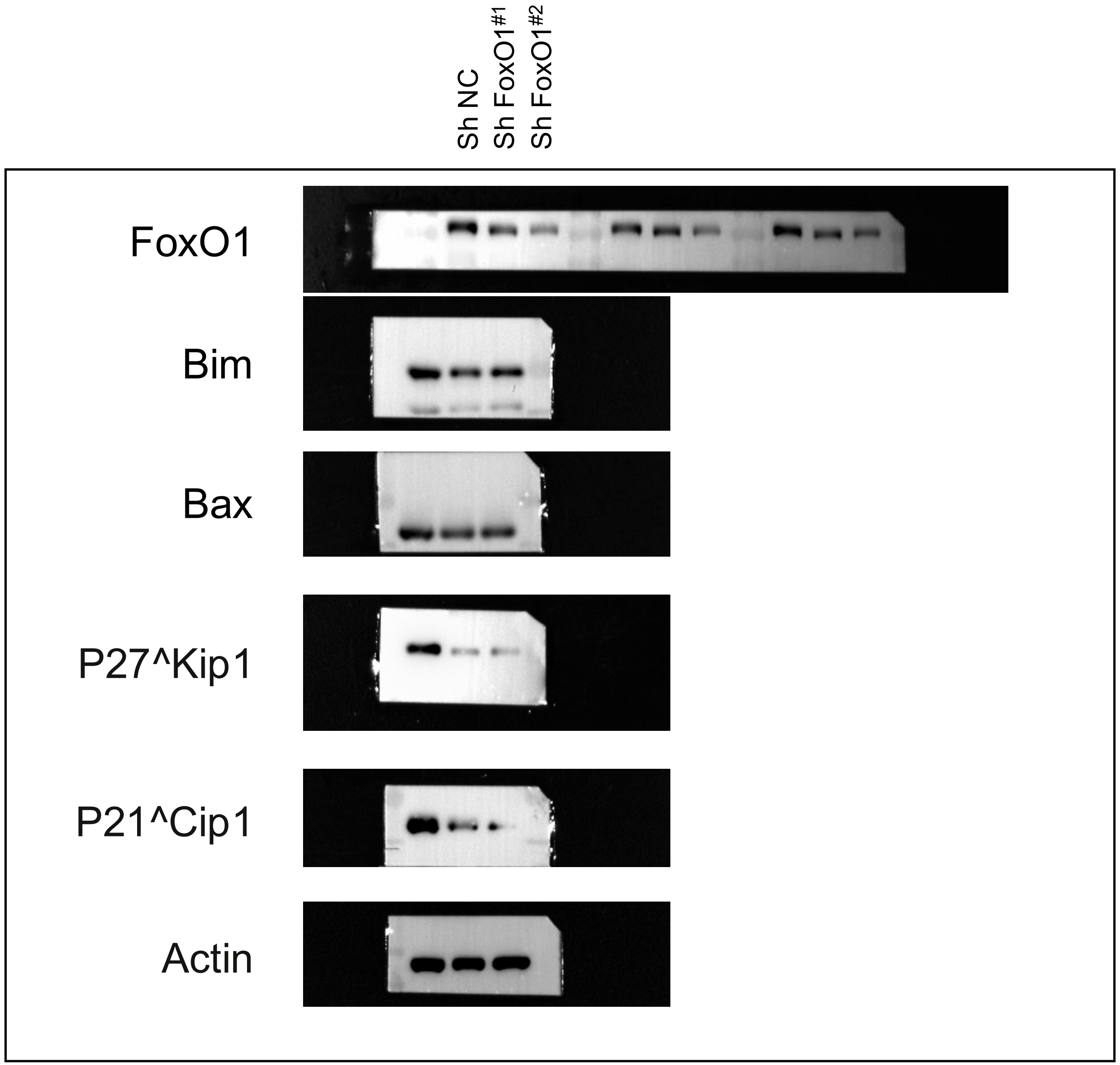
Sh NC
Sh FoxO1#1
Sh FoxO1#2
FoxO1
Bim
Bax
P27^Kip1
P21^Cip1
Actin
